# Macrophage-related immune responses in inner ear: a potential therapeutic target for sensorineural hearing loss

**DOI:** 10.3389/fnins.2023.1339134

**Published:** 2024-01-11

**Authors:** Yu-Chen Liu, Kai Xu

**Affiliations:** ^1^Queen Mary School, Jiangxi Medical College, Nanchang University, Nanchang, China; ^2^Department of Otolaryngology, Head and Neck Surgery, The Second Affiliated Hospital of Nanchang University, Jiangxi Medical College, Nanchang University, Nanchang, China

**Keywords:** sensorineural hearing loss, hair cell, gene therapy, immune response, macrophage

## Abstract

Hearing loss is the most common sensory disorder in human beings. Cochlear sensory cells are the basis of hearing. Cochlear sensory cells suffer from various acute or chronic injuries, such as excessive sound stimulation, ototoxic drugs, and age-related degeneration. In response to these stresses, the cochlea develops an immune response. In recent years, studies have shown that the immune response of the inner ear has been regarded as one of the important pathological mechanisms of inner ear injury. Therapeutic interventions for inflammatory responses can effectively alleviate different types of inner ear injury. As the main immune cells in the inner ear, macrophages are involved in the process of inner ear injury caused by various exogenous factors. However, its specific role in the immune response of the inner ear is still unclear. This review focuses on discusses the dynamic changes of macrophages during different types of inner ear injury, and clarifies the potential role of macrophage-related immune response in inner ear injury.

## 1 Introduction

Hearing loss is one of the most common sensory functional diseases in humans. At present, as many as 1.5 billion people worldwide suffer from hearing loss. Sensorineural hearing loss (SNHL), an important type of hearing loss, resulting from dysfunction of the inner ear, auditory nerve, or the auditory central nervous system. The loss or dysfunction of cochlear sensory cells and spiral ganglion is the main cause of SNHL. Currently, the molecular mechanisms of SNHL has not been fully clarified. Recently, there is increasing evidence that the cochlear immune responses are directly involved in the process of inner ear injury and immune-mediated SNHL has been widely accepted. The inner ear has been recognized as an immune privileged organ due to the blood-labyrinth barrier that separates the cochlear microenvironment from circulation. Harris et al. (1997) observed direct contact between lymphocytes and macrophages in the endolymphatic sac. Fredelius and Rask-Andersen (1990) subsequently reported infiltration of immune cells into the cochlea following noise exposure. In addition, studies have shown the presence of resident immune cells in the cochlea under normal conditions, this phenomenon has attracted renewed interest in inner ear immunity (Sato et al., 2008). These results suggest that the cochlea, like other organs, interacts with the systemic immune system through lymphatic drainage and vascular circulation to fight infection or exogenous injury. In recent years, a variety of inner ear immune cells have been identified, among which macrophages are the most abundant immune cells in the cochlea. Studies have shown that macrophages exist at different stages of cochlear development, and the presence of macrophages in the otic capsule was observed on embryonic day 10 (E10), suggesting that cochlear macrophages may originate in the embryo. In addition, macrophage precursor cells were detected in the mouse cochlea early after birth, and these precursor cells may be an important source of tissue macrophages (Dong et al., 2018). Although the origin and precise functions of cochlear macrophages are not clear, the presence of cochlear macrophages throughout life suggests that they are involved in maintaining cochlear tissue homeostasis. More specifically, cochlear macrophages not only participate in the development of the cochlea and maintain homeostasis, but also play an important role in the local immunity of the cochlea. Macrophage-mediated immune response is involved in a variety of acute and chronic inner ear injury processes, including noise, ototoxic drugs, viral infections, cochlear implant surgery, or age-related hearing loss. After cochlear injury, the number of cochlear macrophages increased significantly, and the morphology and phenotype of macrophages also changed significantly. Studies have shown that cochlear macrophages exhibit different phenotypes in different types of injury. In the acute injury of the cochlea, the increase of cochlear macrophages is mainly derived from monocytes in peripheral blood. However, in the chronic injury of the cochlea, the cochlea is mainly characterized by the differentiation and activation of resident macrophages. The activation of both infiltrating macrophages and resident macrophages can lead to changes in the local internal environment of the cochlea. Infiltrating macrophages can aggravate the occurrence of cochlear inflammation by directly contacting damaged cells or secreting cytokines. However, the exact role of macrophage-mediated immune response in the cochlea injury remains unclear.

In recent years, the immune response of the inner ear has been regarded as one of the important pathological mechanisms of inner ear injury. Therapeutic interventions for inflammatory responses can effectively alleviate different types of inner ear injury. In this review, we will discuss the dynamic changes of macrophages in the process of different types of inner ear injury, and try to clarify the potential role of macrophage-related immune response in inner ear injury.

## 2 Origin of cochlear macrophages

Traditionally, the inner ear has been considered to be an immune privileged organ. However, in recent years, a variety of inner ear immune cells have been identified, among which macrophages are the most abundant immune cells in the cochlea (Hirose et al., 2005; Okano et al., 2008). Macrophages are present at different stages of cochlear development. The presence of macrophages in the otic capsule was observed on embryonic day 10, suggesting that cochlear macrophages may originate from embryos. In the early postnatal period, macrophage precursor cells were detected in the cochlea, which may be an important source of tissue macrophages (Dong et al., 2018). In adult mice, the origin of macrophages in the cochlea is not fully understood. In peripheral tissues, bone marrow-derived precursor cells are the main source of tissue macrophages (Ginhoux and Jung, 2014). Shi (2010) have reported that the cochlear macrophages were differentiated from bone marrow-derived monocytes under steady-state conditions through bone marrow transplantation experiments in irradiated mice. In addition to macrophages, the cochlear immune cell population also includes granulocytes (3.1%), T cells (0.8%), B cells (0.4%), and natural killer cells (3.4%) (Matern et al., 2017). The cochlea of newborn mice has undergone remodeling during development, and the composition of its immune cells has also changed. Identifying the origin of macrophages in different developmental stages of the cochlea is crucial for understanding their role in cochlear homeostasis and disease formation.

## 3 Distribution of cochlear macrophages

**FIGURE 1:**
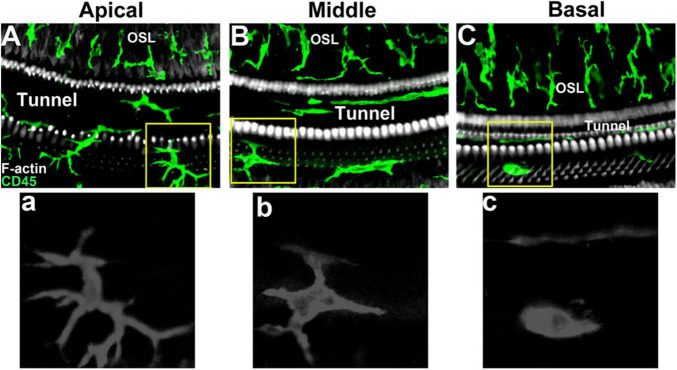
Macrophage morphology in different regions of mouse basilar membrane under steady-state conditions. **(A, a)** Apical turn of basilar membrane. **(B, b)** Middle turn of basilar membrane. **(C, c)** Basal turn of basilar membrane.

Under steady-state conditions, macrophages are mainly distributed in the spiral ligament and stria vascularis of the lateral wall of the cochlea (Sato et al., 2008). In the spiral ligament, the macrophages were irregularly branched and mainly distributed in the lower region of the spiral ligament. This region is rich in type II and type IV fibroblasts, which are prone to pathological changes (Hirose and Liberman, 2003). During acute injury, the number of macrophages in the area adjacent to the scala tympani of the spiral ligament increases sharply (Miyao et al., 2008; Du et al., 2011). In addition, there are also abundant macrophages in the stria vascularis. In the stria vascularis, macrophages are mainly distributed around the capillaries, and the dendritic branches are consistent with the blood vessels, which together with the endothelial cells of the capillaries constitute the blood membrane labyrinth barrier (Zhang et al., 2012). Shi et al. showed that lipopolysaccharide-induced inflammatory response significantly increased vascular permeability and leakage, and destroyed the integrity of the blood membrane labyrinth (Jiang et al., 2019). In addition, macrophages are widely distributed in the cochlear nerve tissue. Near the nerve fibers of the cochlear axis, the dendritic protrusions of macrophages are the same as the nerve fibers. Studies have shown that macrophages in these regions are involved in the development of auditory nerves in addition to the inflammatory response in the cochlea (Brown et al., 2017). In the spiral limbus, macrophages and nerve fibers can pass through the habenula, but do not contact with the inner hair cells (Hirose et al., 2017). In the Rosenthal duct, macrophages are mainly distributed around the spiral ganglion. Dong et al. (2018) analyzed the number of macrophages in this region at different stages of cochlear development. The results showed that the number of macrophages region decreased with age in the spiral ganglion. Macrophages increase significantly when spiral ganglion cells are damaged, and aggregated macrophages can alleviate the damage of spiral ganglion to a certain extent. Under physiological conditions, there are abundant macrophages on the tympanic side under the basilar membrane of the mature cochlea, which are distributed along the basilar membrane from top to bottom. Macrophages are immersed in the tympanic perilymph and in direct contact with basement membrane mesothelial cells. This unique distribution allows it to monitor changes in the basilar membrane and perilymph microenvironment. The number and morphology of macrophages in different turns of the basilar membrane are different (Hu et al., 2018). The macrophages in the apical of the basilar membrane were dendritic, with small cell bodies and long branches (Figure 1A, a). The macrophages near the middle turns were amoeba-like, the cell body became larger, and the branches became shorter and thicker (Figure 1B, b). The macrophages in the basal turns of the basilar membrane were round, with almost no branched protrusions (Figure 1C, c). In addition, there are also some differences in the expression patterns of macrophage immune molecules in different sections of the basilar membrane, suggesting that they play different roles in the immune response (Hashimoto et al., 2005; Yang et al., 2015). Basilar membrane macrophages are closest to sensory cells, so they are more likely to feel the damage of sensory cells. Studies have shown that these macrophages monitor changes in the basilar membrane microenvironment (Zhang et al., 2017).

## 4 Macrophage phenotype in acute cochlear injury

In recent years, there are increasing evidence that the inner ear immune response were involved in the acute injury of sensory epithelial cells caused by a variety of exogenous factors, including noise, ototoxic drugs, viral infection, cochlear implantation, and other injuries (Fujioka et al., 2006; Warchol et al., 2012; Verschuur et al., 2015; Tan et al., 2016). The immune response of the cochlea is a sterile inflammatory response without the participation of pathogens, death of hair cells and secretion of pro-inflammatory factors are one of the main factors that trigger the recruitment and activation of cochlear immune cells. The inflammatory response of the inner ear caused by acute cochlear injury is characterized by the infiltration of monocytes. Peripheral circulating monocytes are the main source of immune cells in the cochlea (Hirose et al., 2005). After cochlear injury, peripheral circulating monocytes enter the cochlea and differentiate into functional macrophages. Yang et al. (2015) observed the infiltration monocytes in the lateral wall of the cochlea after acoustic injury, and the infiltrated monocytes were transformed into macrophages. The activated monocyte-derived macrophages amplify the cochlear inflammatory response through antigen presentation and secretion of inflammatory factors, and participate in the process of sensory epithelial injury (Yang et al., 2015). Excessive noise stimulation can cause damage to hair cells, and the cytokines secreted by disintegrated hair cells and resident cells promote the recruitment of cochlear macrophages to the damaged area for local inflammatory response. In addition, after noise exposure, the expression of cochlear inflammation-related cytokines increased significantly, including chemokines, adhesion molecules, and macrophage migration inhibitory factors (Frye et al., 2019; Landegger et al., 2019). Chemokines are powerful inducers of macrophage activation and migration, and adhesion molecules can mediate macrophage recognition and adhesion. Studies have shown that the activated cochlear macrophages can secrete a variety of cytokines. In the acute phase of the inflammatory response of the inner ear after noise exposure, the expression of tumor necrosis factor-α (TNF-α), interleukin-6 (IL-6), and interleukin-1β (IL-1β) increased sharply, which played an important role in respond to tissue damage (Ladrech et al., 2007; Wakabayashi et al., 2010).

In addition, the recruitment and activation of macrophages were also observed in the ototoxicity model. In the mouse cochlear culture model, the number of macrophages in the sensory epithelial injury area was significantly increased after kanamycin treatment. The activated macrophages can quickly migrate to the damaged cells and protrude to engulf the damaged sensory cells (Hirose et al., 2017). Sato et al. (2010) also observed monocyte infiltration and macrophage activation in the basilar membrane and spiral ganglion cell injury area in an mouse ototoxic model. Lenoir et al. showed that macrophages are involved in amikacin-induced sensory epithelial injury in rats, and early anti-inflammatory treatment may promote the survival of supporting cells (Ladrech et al., 2007). Kaur et al. (2015b) constructed a diphtheria toxin receptor knock-in mouse model, and observed a large number of hair cells loss and the supporting cells were not damaged after injection of diphtheria toxin, accompany with the number of basement membrane macrophages increased significantly. It indicates that the pro-inflammatory signal released by the damage of hair cells is sufficient to activate the basement membrane macrophages, even if the macrophages cannot directly contact with the damaged hair cells. However, many macrophages appeared to actively phagocytose hair cell debris in the damaged area of sensory cells in the utricle of mice, suggesting that macrophages may be directly involved in the corpse clearance process of mammalian vestibular organs (Kaur et al., 2015a). In addition, cochlear implantation can cause transient damage to the cochlea and nerves. The electrode implantation was simulated on the animal model, and the inflammatory reaction of macrophages in the electrode injury area was involved in the repair and healing of the wound (Okayasu et al., 2019, 2020). In general, macrophage related immune response of inner ear has been regarded as one of the important pathological mechanisms of acute inner ear injury.

## 5 Macrophage phenotype in Chronic cochlear injury

At present, the understanding of macrophage-related inflammatory response mainly comes from the study of acute inner ear injury. The infiltration and activation of cochlear macrophages play a crucial role in the process of acute inner ear injury. In recent years, researchers have also observed the participation of macrophages in chronic cochlear injury models. There are many differences in the pathological mechanisms of acute and chronic cochlear injury. The degeneration of sensory epithelial cells progresses rapidly during acute injury, followed by monocyte infiltration and macrophage activation. On the contrary, the damage of cochlear sensory epithelium in chronic injury progresses slowly, and the cochlea shows chronic inflammatory response. In this process, the activation of cochlea resident macrophages was mainly observed, and macrophages differentiated from infiltrating monocytes into the cochlea are rarely observed. Hu et al. have reported the dynamic activation of basilar membrane resident macrophages response to chronic hair cell degeneration, and tissue-resident macrophages, not infiltrated monocytes, are the main executors of immune response (Frye et al., 2017). Notably, activation of basilar membrane resident macrophage precedes hair cell degeneration, and the activity of macrophages is maintained until sensory cell are completely degraded. In the cochlear specimens of the elderly, the number of activated macrophages around the auditory nerve was increased, and macrophages surrounded the myelinated axon fibers, suggesting that macrophages were involved in the degeneration of the auditory nerve (Fischer et al., 2020). In addition, after low-intensity noise stimulation, mice showed temporary hearing impairment and no death of sensory epithelial cells was observed. The number of macrophages in the basilar membrane and spiral limbus was still significantly increased, suggesting that macrophages could monitor the changes of cochlear microenvironment in the early stage of sensory epithelial injury or before death (Frye et al., 2018). Our recent study found that macrophage activation is involved in the pathological process of sensory cell and spiral ganglion injury induced by GJB2 gene knockout in mice, and CX3CL1-CX3CR1 signaling axis is involved in regulating the recruitment of macrophages in the inner ear (Xu et al., 2020). Previous studies have reported that acoustic injury causes the infiltration of circulating leukocytes into the cochlea. Zhang et al. (2021) revealed that CX3CR1 is involved in regulating the infiltration of neutrophils in the cochlea, lack of CX3CR1 results in the augmentation of neutrophil infiltration into cochlear tissues after acoustic trauma. Sato et al. (2010) pointed out that CX3CR1-deficient cochlear macrophages exacerbate kanamycin induced damage, suggest that CX3CR1 plays a role in modulating the cochlear macrophages after kanamycin ototoxicity. In addition, disruption of CX3CL1-CX3CR1 signaling reduced macrophage infiltration into both the sensory epithelium and spiral ganglion, and also resulted in diminished survival of spiral ganglion neurons (Kaur et al., 2015b). Jabba et al. (2006) also observed macrophage infiltration in the stria vascularis of the Pendred syndrome mouse model. Chronic inflammation is a crucial contributor to various age-related disease and natural processes in aging tissue, including the cochlear and nervous system. Verschuur et al. (2012) reported that the hearing threshold in the population was directly related to the key serum biomarkers of low-grade inflammation. Inflammatory cytokines such as IL-1α, IL-2, TNF-α and NF-κB play an important role in the initiation and regulation of chronic inflammation in the inner ear (Satoh et al., 2003, 2006). These data support that the chronic inflammation plays a role in mechanisms of age-related hearing loss, and there is still little known about inflammation in the ageing cochlea.

## 6 Function of macrophages in cochlear injury

### 6.1 Phagocytosis

Phagocytosis is one of the important functions of macrophages. Like macrophages in other organs, cochlear macrophages also have phagocytic function. They continuously monitor the dynamic changes of the cochlear microenvironment. Frye et al. observed macrophage phagocytosis of red blood cells on the surface of the mouse cochlea (Hu et al., 2018). Sensory cells of the cochlea are susceptible to external injury factors, resulting in apoptosis, necrosis, or mixed-mode death (Ylikoski et al., 2002; Bohne et al., 2007). The phagocytosis of macrophages is more obvious in the cochlear injury area. *In vitro* experiments by Hirose et al. (2017) observed that in the kanamycin-induced cochlear basement membrane injury model, activated macrophages can quickly migrate to the vicinity of damaged hair cells and protrude to phagocytose damaged cells. Kaur et al. (2015a) reported that macrophages were observed to be involved in the phagocytosis and clearance of damaged cell debris in a mouse utricle sensory cell injury model induced by diphtheria toxin targeting. However, the Corti organ of the mature cochlea lacks macrophages under steady-state conditions. There is no evidence that macrophages can directly enter the Corti organ damage area and directly participate in the cleaning of damaged cells in the *in vivo* model. Studies have reported that Deiter cells are involved in the phagocytosis of damaged sensory cells and the filling of missing sensory epithelial cells (Abrashkin et al., 2006). As immune cells adjacent to the organ of Corti, the recruitment of macrophages on the tympanic side of the basilar membrane in the damaged area of the organ of Corti may play a role by secreting cytokines.

### 6.2 Secretion of inflammation-related cytokines

In recent years, a series of studies have shown that increased expression of inflammation-related cytokines in the cochlea has been detected in many different injury models (Cho et al., 2004; Gratton et al., 2011; Cai et al., 2014). In the cochlea, the interaction between inflammatory factors and macrophages is an important part of the cochlear immune response. Macrophages are involved in the mobilization and amplification of inflammatory response. When stimulated by exogenous stress, cytokines in the cochlea recruit macrophages to damaged tissues, and adhesion molecules promote macrophages to move slowly to the place where resident cells secrete cytokines. After acoustic injury, the expression of intercellular adhesion molecular-1 (ICAM-1) in the cochlea increased, and blocking ICAM-1 can inhibit the recruitment of macrophages and reduce noise-induced cochlear injury (Seidman et al., 2009). In the acute injury phase of noise stimulation, monocytes enter the cochlea and differentiate into macrophages, which can engulf and secrete more molecules, thereby mobilizing further immune response. After noise stimulation, the expression of inflammation-related cytokines in the cochlea, including intercellular adhesion molecule-1, TNF-α, chemokine CCL2, interleukin-6, and interleukin-1β, increased sharply after noise exposure and played an important role in the inflammatory response (Fujioka et al., 2006; Tan et al., 2016; Landegger et al., 2019). The application of RNA sequencing technology to the overall analysis of the transcriptional level of the cochlea under various pathological conditions has basically clarified the participation of a large number of immune-related molecules and multiple immune-related pathways (Patel et al., 2013; Yang et al., 2016). In addition, chemokine-chemokine receptor interactions, complement cascades, NOD-like receptor signaling, and TLR signaling are all involved in the immune response of the cochlea (Sato et al., 2008; Vethanayagam et al., 2016). Immune cells are the main source of immune-related cytokines. Pressure stimulates endothelial cells to present cell adhesion molecules and recruit macrophages to damaged tissues. Macrophages can engulf and secrete more immune factors, thereby mobilizing further immune response. Toll-like receptor 4 (TLR4) is a member of the pattern recognition receptor (PRR) family. Tlr4 deficiency inhibits the expression of major histocompatibility complex class II (MHC-II) in macrophages and reduces the antigen presentation activity of macrophages. However, the precise contribution of immune cells during inner ear injury remains unclear.

### 6.3 Antigen presenting

Under steady-state conditions, there are abundant immune cell populations in the cochlea, including macrophages, granulocytes, T cells, B cells and natural killer cells (Matern et al., 2017). Antigen presentation is an important part of immune activity, which occurs in the initial stage of immune response. It refers to the process of antigen presenting cells ingesting antigens to be processed, presenting on the surface of presenting cells in the form of immune peptides after a series of processing, and being recognized by immune cells to activate immune active cells (Steinman, 1991; Unanue, 1984). The main antigen-presenting cells include dendritic cells, macrophages, B lymphocytes and monocytes. Under steady-state conditions, macrophages in the cochlea express antigen-presenting molecules such as MHC II and CIITA. After excessive noise stimulation, a significant increase in activated macrophages was detected in the damaged area of cochlear sensory cells, and the expression of MHC II and CIITA in activated macrophages was significantly increased (Yang et al., 2015). In addition, the number of T cells is also increasing, and the increase of T cells indicates the activation of adaptive immune response. Our recent study found that in the model of sensory epithelial injury induced by GJB2 gene in targeted knockout mice, activated macrophages expressed MHC II molecules, and the number of MHC II positive cells increased significantly, suggesting that activated macrophages have potential antigen presentation function (Xu et al., 2020).

## 7 Application of anti-inflammatory therapy in the inner ear

In recent years, studies have shown that the treatment of inflammatory response can effectively alleviate different types of inner ear injury. Okano et al. reported that blocking IL-6 with anti-IL-6 antibody can improve the hearing of noise-exposed mice at a specific frequency. Histological analysis showed that the number of activated macrophages in the spiral ganglion region was significantly reduced and the damage of the spiral ganglion was significantly improved (Wakabayashi et al., 2010). Hu et al. showed that knockout of TLR4 gene can inhibit the function of macrophages in the inner ear. After noise stimulation, the hearing loss and hair cell death of TLR4 knockout mice were significantly reduced compared with wild-type mice (Vethanayagam et al., 2016). Mizushima et al. (2017) used the macrophage scavenger clodronate liposomes. The intervention of clodronate liposomes can significantly ameliorated noise-induced hearing loss in mice and protect the loss of outer hair cells (Mizushima et al., 2017). CCL2 and its main receptor CCR2 are important chemokines of monocytes. Hirose et al. reported that after noise stimulation, the hearing loss and hair cell death of CCR2 knockout mice were significantly reduced compared with wild-type mice. However, there was no significant changes in macrophage migration were observed, suggesting that CCR2 receptor is not necessary for situational cell migration (Sautter et al., 2006). However, the study of Kaur et al. (2015b) showed that knockout of CX3CR1 could significantly inhibit the recruitment of macrophages, and the damage of spiral ganglion in CX3CR1 knockout group was aggravated, suggesting that the recruited macrophages could participate in promoting the survival of spiral ganglion. The chemokine CX3CL1 acts on the CX3CR1 receptor on the surface of macrophages to regulate macrophages, which has been reported in adipose tissue and skeletal system (Han et al., 2014; Shen et al., 2018). Sun et al. (2015) reported that inhibition of macrophage activation can reduce neomycin-induced hair cell loss and improve the hearing function of neomycin-treated mice. Benseler et al. used IL-1 blockers to significantly improve hearing in patients with Muckle-Wells syndrome (MWS), and the degree of hearing improvement was related to the time of treatment initiation (Kuemmerle-Deschner et al., 2015). Glucocorticoids have many effects such as anti-inflammatory, antitoxin and immune regulation. Systemic or local application of glucocorticoids is the main method for the treatment of SNHL, including sudden sensorineural hearing loss, noise-induced hearing loss, and autoimmune inner ear disease. Intratympanic injection of glucocorticoids can partially improve noise-induced hearing impairment and reduce hair cell apoptosis (Ozdogan et al., 2012; Müller et al., 2017). In recent years, steroids combined with other treatments is also a new treatment strategy. Han et al. (2020) showed that for some patients with refractory sudden deafness, nimodipine combined with steroids had better hearing improvement than single steroid therapy (Han et al., 2020). Prostaglandin E1 combined with glucocorticoid is recommended for the treatment of sudden sensorineural hearing loss patients with severe hearing loss in Japan (Kitoh et al., 2020). Eastwood et al. (2010) showed that injection of glucocorticoids through the round window can improve the high-frequency hearing loss caused by cochlear implantation injury. Ahmadi et al. (2019) reported that hydrogel-loaded dexamethasone can significantly protect the auditory nerve injury caused by cochlear implantation, but the specific mechanism is still unclear. The application of targeted gene therapy has opened up a new way for the treatment of inner ear diseases, through a variety of ways to achieve the expression of exogenous genes in the inner ear. Gene therapy focuses on the reconstruction of genetic materials through repair or gene modification to treat diseases, which is currently the most promising treatment strategy for genetic diseases (Jiang et al., 2023). Therefore, gene therapy based on immune response may be an important potential therapeutic target for sensorineural hearing loss.

## 8 Summary

**FIGURE 2:**
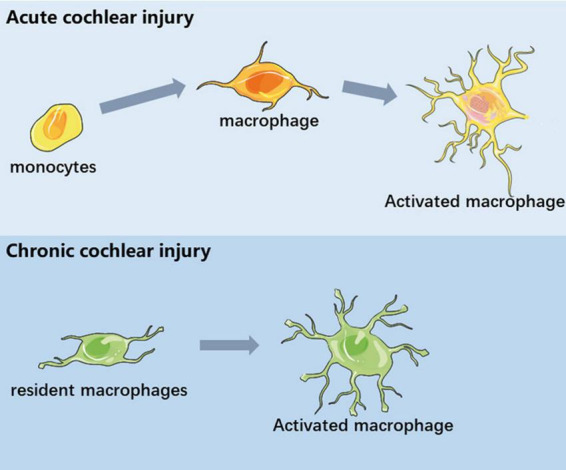
Schematic diagram of macrophages in different inner ear injuries. In acute cochlear injury, circulating monocytes infiltrated the cochlea and differentiate into functional macrophages. In chronic cochlear injury, the activation of basilar membrane resident macrophages is involved in the process of sensory epithelial injury.

In this review, we discuss the immune response and inflammatory activity after cochlear injury. Under steady-state conditions, the cochlea contains a variety of immune cells, of which tissue macrophages are the most abundant. The specific function of resident macrophages in the cochlea and the difference from circulating infiltrating macrophages need to be further explored. In different types of cochlear injury models, tissue macrophages exhibit a variety of morphological and gene expression patterns, indicating that they are in different functional states. The cochlea recruits circulating monocytes when it encounters external pressure, and these immune cells participate in the cochlear immune response together with cochlear resident macrophages (Figure 2). In addition, macrophage-related immune response not only plays a role in acute cochlear injury, but also studies have proposed the concept of inflammation as a mechanism of aging and age-related hearing loss. Macrophage-related immune response in the inner ear has been regarded as one of the important pathological mechanisms of inner ear injury. The role of macrophages in maintaining cochlear homeostasis and participating in inflammatory regulation is becoming more and more clear. In recent years, some progress has been made in the treatment of multiple models of inflammatory response. Macrophage-related inflammation will be used as a target for the treatment of inner ear injury. In the future, we look forward to the discovery of more targeted drugs based on inner ear immune-related molecules.

## Author contributions

Y-CL: Writing – original draft. KX: Writing – review & editing.
